# Menstrual blood-derived mesenchymal stromal cells efficiently ameliorate experimental autoimmune encephalomyelitis by inhibiting T cell activation in mice

**DOI:** 10.1186/s13287-022-02838-8

**Published:** 2022-04-11

**Authors:** Yonghai Li, Haiyao Gao, Tobias M. Brunner, Xiaoxi Hu, Yushan Yan, Yanli Liu, Liang Qiao, Peihua Wu, Meng Li, Qing Liu, Fen Yang, Juntang Lin, Max Löhning, Ping Shen

**Affiliations:** 1 Stem Cell and Biotherapy Engineering Research Center of Henan Province, School of Life Sciences and Technology, Xinxiang Medical University, Xinxiang, 453003 China; 2 Pitzer Laboratory of Osteoarthritis Research, German Rheumatism Research Center (DRFZ), Leibniz Institute, 10117 Berlin, Germany; 3 Experimental Immunology and Osteoarthritis Research, Department of Rheumatology and Clinical Immunology, Charité – Universitätsmedizin Berlin, Corporate Member of Freie Universität Berlin and Humboldt-Universität zu Berlin, 10117 Berlin, Germany; 4 Henan Key Lab of Biological Psychiatry, The Second Affiliated Hospital of Xinxiang Medical University, Xinxiang, 453002, China; 5 School of Medical Engineering, Xinxiang Medical University, Xinxiang, 453003 China

**Keywords:** Menstrual blood-derived mesenchymal stromal cells, Ready-to-use allo-MSCs, Multiple sclerosis, Experimental autoimmune encephalomyelitis, T cell activation suppression, Antigen-presenting cells

## Abstract

**Background:**

Immunosuppressive properties grant mesenchymal stromal cells (MSCs) promising potential for treating autoimmune diseases. As autologous MSCs suffer from limited availability, the readily available allogeneic MSCs isolated from menstrual blood (MB-MSCs) donated by young, healthy individuals offer great potential. Here, we evaluate the therapeutic potential of MB-MSCs as ready-to-use allo-MSCs in multiple sclerosis, an autoimmune disease developed by the activation of myelin sheath-reactive Th1 and Th17 cells, by application in its animal model experimental autoimmune encephalomyelitis (EAE).

**Methods:**

We assessed the therapeutic effect of MB-MSCs transplanted via either intravenous (i.v.) or intraperitoneal (i.p.) route in EAE in comparison with umbilical cord-derived MSCs (UC-MSCs). We used histology to assess myelin sheath integrity and infiltrated immune cells in CNS and flow cytometry to evaluate EAE-associated inflammatory T cells and antigen-presenting cells in lymphoid organs.

**Results:**

We observed disease-ameliorating effects of MB-MSCs when transplanted at various stages of EAE (day − 1, 6, 10, and 19), via either i.v. or i.p. route, with a potency comparable to UC-MSCs. We observed reduced Th1 and Th17 cell responses in mice that had received MB-MSCs via either i.v. or i.p. injection. The repressed Th1 and Th17 cell responses were associated with a reduced frequency of plasmacytoid dendritic cells (pDCs) and a suppressed co-stimulatory capacity of pDCs, cDCs, and B cells.

**Conclusions:**

Our data demonstrate that the readily available MB-MSCs significantly reduced the disease severity of EAE upon transplantation. Thus, they have the potential to be developed as ready-to-use allo-MSCs in MS-related inflammation.

**Graphical abstract:**

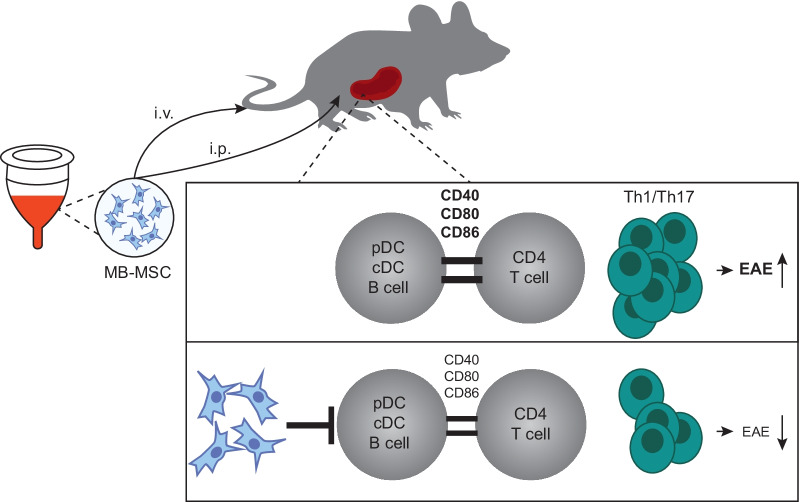

**Supplementary Information:**

The online version contains supplementary material available at 10.1186/s13287-022-02838-8.

## Background

Mesenchymal stromal cells (MSCs) are nonhematopoietic progenitor cells primarily located in umbilical cord, bone marrow, and adipose tissue. MSCs have the potential to differentiate into multiple lineages, including chondrocytes, adipocytes, as well as osteoblasts [1, 2], and are thus considered a promising tool for cell-based regenerative therapy [3]. Data from preclinical studies demonstrate that an intrinsic immunosuppressive capacity of MSCs constitutes a major part of their therapeutic effects [4, 5]. In addition, their low immunogenicity, due to a modest expression of MHC-I and a complete lack of MHC-II [6, 7] and co-stimulatory molecules, helps them to avoid immune surveillance [8].

While MSCs can be isolated from patients and re-applied as autologous MSCs to avoid immune rejection, certain types of diseases restrict patients from supplying MSCs by themselves. For example, myelofibrosis impairs the quality of bone marrow-derived MSCs (BM-MSCs) [9], and systemic diseases, such as diabetes, RA, and SLE alter the intrinsic properties of MSCs [10–12]. Patient age also heavily impacts the availability and functionality of MSCs, as it is generally difficult to procure and extract MSCs from infants, while MSCs from the elderly display decreased biological activity and hence deficits in differentiation and immunoregulation potential [13–16]. In addition, acute diseases such as stroke and myocardial infarction do not allow enough time to extract and expand autologous MSCs and instead need ready-to-use products. Allo-MSCs from young healthy donors are a plausible approach to overcome these difficulties. Indeed, transplantation of human BM-MSCs has been approved for the management of refractory acute GVHD in children unresponsive to systemic steroid therapies. Currently, they are also tested in clinical trials for Crohn's disease [17], GVHD [18, 19], epidermolysis bullosa [20], COVID-19 [21], and for the repair of heart tissue following myocardial infarction [22]. So far, initial results showed that they are well tolerated.

Menstrual blood, monthly shed by women above twelve to fifteen years of age, contains self-renewing stromal cells. In 2007, Meng et al. first isolated MB-MSCs and confirmed their MSC properties, including MSC surface marker expression, self-renewal, and trilineage differentiation potential [23]. MB-MSCs can be collected regularly and non-invasively, providing important potential for biobanking. With regard to stromal cell biological properties, MB-MSCs are comparable to other MSCs with a high proliferation rate [23]. Recent work from the group of Li and her collaboration partners has essentially proven the safety and efficiency of MB-MSCs as allo-MSCs in treating COVID-19 patients [24]. To date, no study has evaluated the therapeutic effects of MB-MSCs in preclinical models of MS.

MS is a neurodegenerative autoimmune disease affecting the central nervous system [25]. It is the most common disabling neurological disorder in young adults and the third largest cause of significant disability for adults between 20 and 40 years [26, 27]. Despite the use of new immunomodulatory agents such as Natalizumab, most patients eventually enter relapsing–remitting phases, accompanied by vicious immunodeficiency complications due to unspecific immune cell depletion or inhibition [28]. Moreover, these therapeutic agents lack the capacity to promote remyelination and therefore the potential for repairing patients´ neurological function [29, 30].

Aiming to overcome these critical drawbacks of current MS therapies, we evaluated the therapeutic effects of MB-MSCs—which hold, among other benefits of MSCs, the advantage of non-invasive and periodical acquisition—in the murine MS model EAE. We first show the disease-ameliorating function of MB-MSCs when transplanted at various stages of EAE and via both intravenous and intraperitoneal routes. Further, we found that the disease-ameliorating effect of MB-MSCs was associated with suppressed inflammatory immune responses in both peripheral lymphoid organs and the CNS, represented by repressed APC activity, lower frequencies of Th1 and Th17 cells, and fewer lymphocytes infiltrating the CNS. Lastly, we show that MB-MSCs had therapeutic effects similar to UC-MSCs. Thus, MB-MSCs have the potential to be developed as a read-to-use allo-MSC therapeutic agent.

## Methods

### Mice and EAE induction

We used female C57BL/6 mice, 8–12 weeks of age at the start of experiments. Mice were housed in the animal facilities of Xinxiang Medical University and German Rheumatism Research Center (DRFZ) Berlin. All experimental procedures were approved by the ethics committee of Xinxiang Medical University, China, and the Landesamt für Gesundheit und Soziales, Berlin, Germany. EAE was induced by subcutaneous immunization with 50 µg MOG_35-55_ peptide emulsified in Complete Freund’s Adjuvant containing 400 µg mycobacteria and intravenous injection of two doses pertussis toxin on day 0 and 2 after immunization. Clinical signs of EAE were assessed with the following 0–6 scoring system: 0—no signs; 1—flaccid tail; 2—impaired righting reflex; 3—paralysis of one hind limb; 4—paralysis of both hind limbs; 5—paralysis of both hind limbs; severe weight loss (20%) and reduced mobility (mice with a score of 5 were killed); and 6—moribund or dead. (In the case of moribund, mouse were killed.) EAE experiments were scored in a blinded manner.

### Isolation and culture of MB-MSCs

Menstrual blood samples were donated by healthy volunteers during the first few days of menses. Upon collection, blood samples were subjected to standard Ficoll gradient centrifugation. The mononuclear cells at the interlayer were harvested together with the suspending deciduous endometrium and plated in T25 flasks with complete DMEM medium supplemented with 15% FBS and 100 U/ml penicillin/streptomycin. After two days of culture, culture medium was replaced to remove non-adherent cells. MB-MSCs remained as adherent cells grown into visible colonies. Medium was renewed every 3 days, and MB-MSCs were subcultured to new flasks when they reached 80% confluence (P0). Cells were then either directly used for experiments or cultured further till passage 3 and frozen in liquid nitrogen. The entire procedures were performed with consent of the donors and approved by the Ethics Committee of Xinxiang Medical University, China.

### MB-MSC transplantation

Four days before transplantation, P3 MB-MSCs were thawed and cultured in T75 flasks. On the day of transplantation, usually a 70–80% cell confluency was reached and MB-MSCs were detached, counted, and diluted to a final concentration of 3.3 × 10^6^/mL. 1 × 10^6^ cells in 300 µl were injected intravenously (very slowly) or intraperitoneally per mouse. For MB-MSCs tracking, cells were labelled with CFSE before transplantation according to manufacturer’s instructions.

### Cell staining for flow cytometry

For surface stainings, MB-MSCs of P0 to P10 were first incubated with IVIG (intravenous immunoglobulin, a blood product prepared from the serum of between 1000 and 15,000 donors per batch) for 15 min to block unspecific binding. Cells were stained with Zombie Aqua to exclude dead cells combined with directly conjugated antibodies targeting CD9, CD29, CD44, CD73, CD90, CD105, CD166, CD34, CD45, CD14, CD80, CD86, HLA-ABC, HLA-DR at 4 °C for 20 min. Mouse splenocytes were first incubated with anti-FcγR blocking antibody for 15 min, followed by staining with antibodies against CD4, BB20, CD11c, PDCA-1, CD11b, Ly6C, Ly6G, CD40, CD70, CD80, CD86, MHCII, OX40L, and Zombie Aqua at 4 °C for 20 min. For transcription factor stainings, cells were fixed and permeabilized with FOXP3 Fixation/Permeabilization Buffer from eBioscience and subsequently stained with anti-T-bet and anti-RORγt antibodies for 30 min at 4 °C. For intracellular cytokine stainings, splenocytes were plated at 4 × 10^6^ cells per well in 48-well plates and stimulated with 20 µg/ml MOG peptide in the presence of Brefeldin A. After five hours of stimulation, cells were harvested, subjected to surface blocking and staining, fixed with 4% formalin for 15 min, and stained with anti-IFNγ, anti-IL-17, anti-GM-CSF, anti-TNFα, and anti-CD40L antibodies for 30 min. Stained cells were acquired on a FACS Canto flow cytometer and analyzed with FlowJo.

### Histological staining

Spines were fixed with 4% formaldehyde for 48 h, decalcified with EDTA for 1 week, followed by paraffin embedding and sectioning. Spine sections were first deparaffinized, then rehydrated with distilled water, and were subsequently incubated with primary antibodies specific against CD3 (N1580, Dako, Glostrup), F4/80 (clone BM8, eBioscience), or Iba-1 (cat number 019-19741, Wako) for 30 min, followed by staining with biotinylated donkey anti-rat or donkey anti-rabbit secondary antibodies (Dianova). A streptavidin-AP kit was used for detection. For Fast Blue staining, spine sections were stained with Luxol Fast Blue solution at 58 °C overnight. After washing with 95% alcohol and distilled water, sections were stained with a lithium carbonate solution followed by an additional round of washing with 70% alcohol and distilled water.

### Statistical analysis

Statistical analysis was performed using GraphPad Prism (v5.02 and v7). All data are presented as Mean ± SEM and were analyzed by *t* test and one-way ANOVA with significance measured at *P < 0.05, **P < 0.01, or ***P < 0.001.

## Results

### Passage four-MB-MSCs were chosen for transplantation

**Fig. 1:**
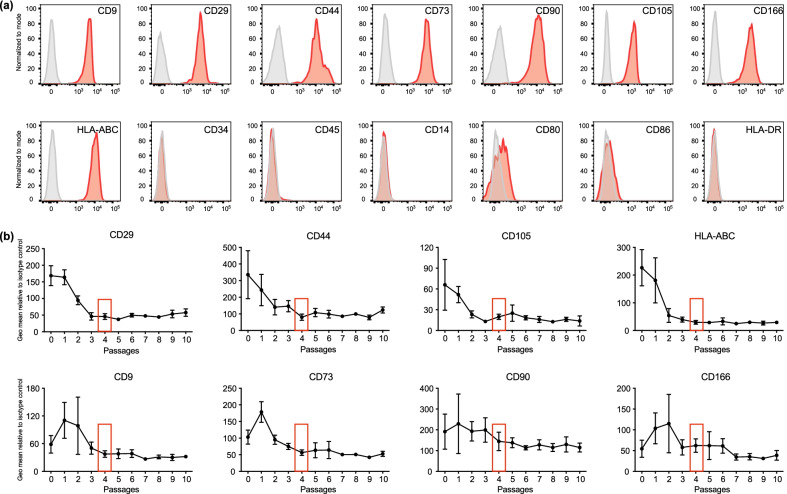
MB-MSC phenotype and its passaging-associated alterations. MB-MSCs were isolated and maintained in monolayer culture. **a** Exemplary FACS plots with passage 4 (P4) MB-MSCs illustrate that they are positive for classical MSC surface markers CD9, CD29, CD44, CD73, CD90, CD105, CD166, and HLA-ABC, and negative for hematopoietic cell markers CD34, CD45, CD14, CD80, CD86, and HLA-DR. **b** The expression level (geometric mean) of each individual MSC surface marker at the end of each passage was measured by FACS and normalized to its corresponding isotype control staining. Plots show the expression kinetics of each surface marker from passage 0 to passage 10. *n* = 3

It is well recognized that extended expansion in vitro can compromise the functionality of MSCs. Our previous study demonstrated that the proliferative rate of MB-MSCs decreases with increasing passages [31]. To gain a better understanding of the phenotypic changes that MB-MSCs undergo during passaging, we monitored the surface phenotype of MB-MSCs during a 10-passage culture period via flow cytometry. Eight MSC-specific markers (CD9, CD29, CD44, CD73, CD90, CD105, CD166, and HLA-ABC) were evaluated, and six hematopoietic cell markers (CD34, CD45, CD14, CD80, CD86, and HLA-DR) were included to assess purity. The exemplary FACS plots of the surface markers using passage four (P4) MB-MSCs illustrate their appropriate MSC phenotype (Fig. 1a). Moreover, during the 10-passage follow-up examination, we observed that the expression level of MSC surface markers declined from P0 or P1 and stabilized at P3 or P4 (Fig. 1b). Particularly, since the expression level of HLA-ABC correlates with their immunogenicity [32] and P4 was the earliest passage with reduced HLA-ABC expression, we decided to use P4 MB-MSCs in the following experiments. As freshly thawed MSCs have compromised bioenergetics with reduced oxidative phosphorylation activity and ATP production, and also lower therapeutic efficiency [33–35], which can be rescued by a 24-h or longer culture [36], we froze P3 MB-MSCs as stock in liquid nitrogen and cultured them for one more passage for transplantation experiments. Thus, we used P4 MB-MSCs freshly harvested from cell culture as study material throughout this study.

### MB-MSCs ameliorate EAE severity when given intravenously on day 6 after EAE induction

**Fig. 2:**
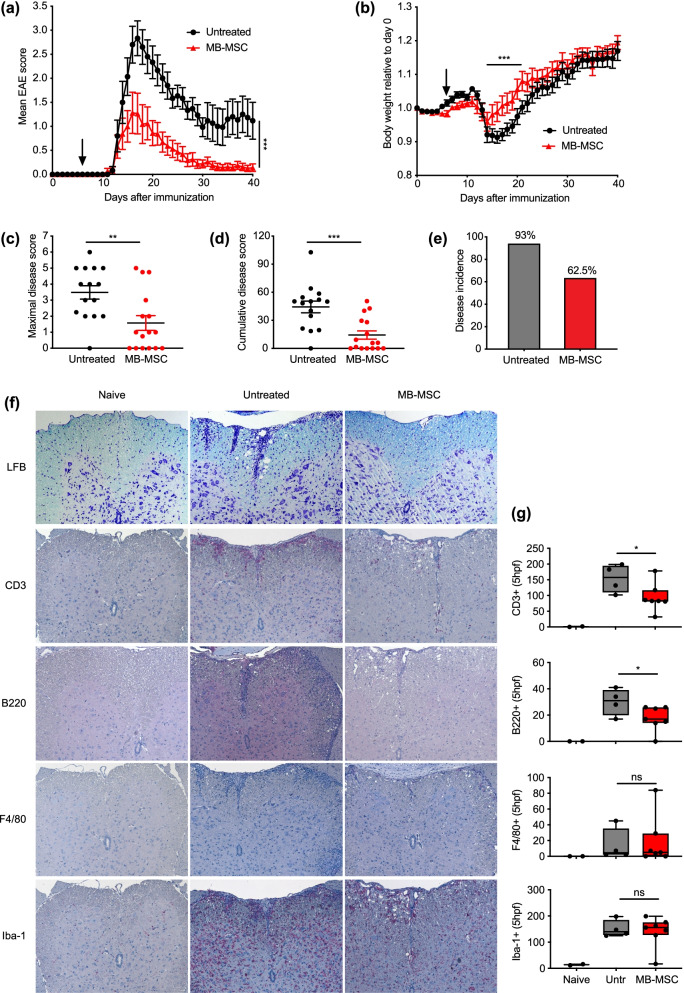
MB-MSC transplantation on day 6 after EAE induction ameliorates EAE. EAE was induced in mice, and 6 days later, P4 MB-MSCs were transplanted via the tail vein (1 × 10^6^/mouse). Mice that received only PBS served as untreated controls. **a** EAE disease severity and the change of body weight (**b**) were monitored daily thereafter. **c**–**e** Maximal disease scores, cumulative disease scores, and disease incidence rates were analyzed. Pool of two experiments. *n* = 15 or 16 per group. **f**, **g** Histological analysis of spinal cords collected on day 19 after EAE induction shown with representative stainings for Fast Blue, CD3, B220, F4/80, and Iba-1, and quantitative plots for CNS-infiltrating and activated immune cells

To investigate whether MB-MSCs can modify the disease progression of EAE, we first induced EAE and six days later, when T cell activity in the draining lymph nodes reached its maximum (Additional file 1: Fig. S1a), transplanted one million MB-MSCs intravenously (i.v.) or injected PBS as control. We observed a remarkable suppressive effect of MB-MSCs on EAE development (Fig. 2a) and on body weight loss (Fig. 2b). Further, MB-MSC transplantation significantly reduced the maximal and the cumulative disease scores of the treated mice (Fig. 2c, d). Notably, MB-MSC transplantation also reduced the disease incidence from 93 to 62.5% (Fig. 2e). Hence, these data collectively demonstrate that MB-MSC transplantation significantly reduces EAE severity.

In EAE as well as in MS, the infiltration of lymphocytes into the CNS—where they cause inflammatory demyelinating lesions—is at the root of the development of clinical symptoms. We assessed how MB-MSC transplantation influenced the demyelination and infiltration of immune cells into the CNS by performing histological analysis of spinal cords taken from naïve, MB-MSC-transplanted, and untreated control mice at the peak of disease. Spines of naïve mice had intact myelin sheath and few T cells, B cells, and macrophages, and most of the microglia cells remained inactive. As expected, in the spines of untreated mice, myelin sheath was largely interrupted and there was a massive influx of T cells, B cells, and macrophages; the resident microglia cells were strongly activated. In contrast, MB-MSC transplantation suppressed the degradation of myelin sheath and significantly reduced the infiltration of CD3^+^ T and B220^+^ B cells, although MB-MSCs had little influence on the infiltration of F4/80 + macrophages and the activation of microglia cells indicated by Iba-1 (Fig. 2f, g). Thus, MB-MSC transplantation reduced the demyelination and inflammation in the CNS.

### MB-MSCs reduce disease severity when transplanted at various stages of EAE

**Fig. 3:**
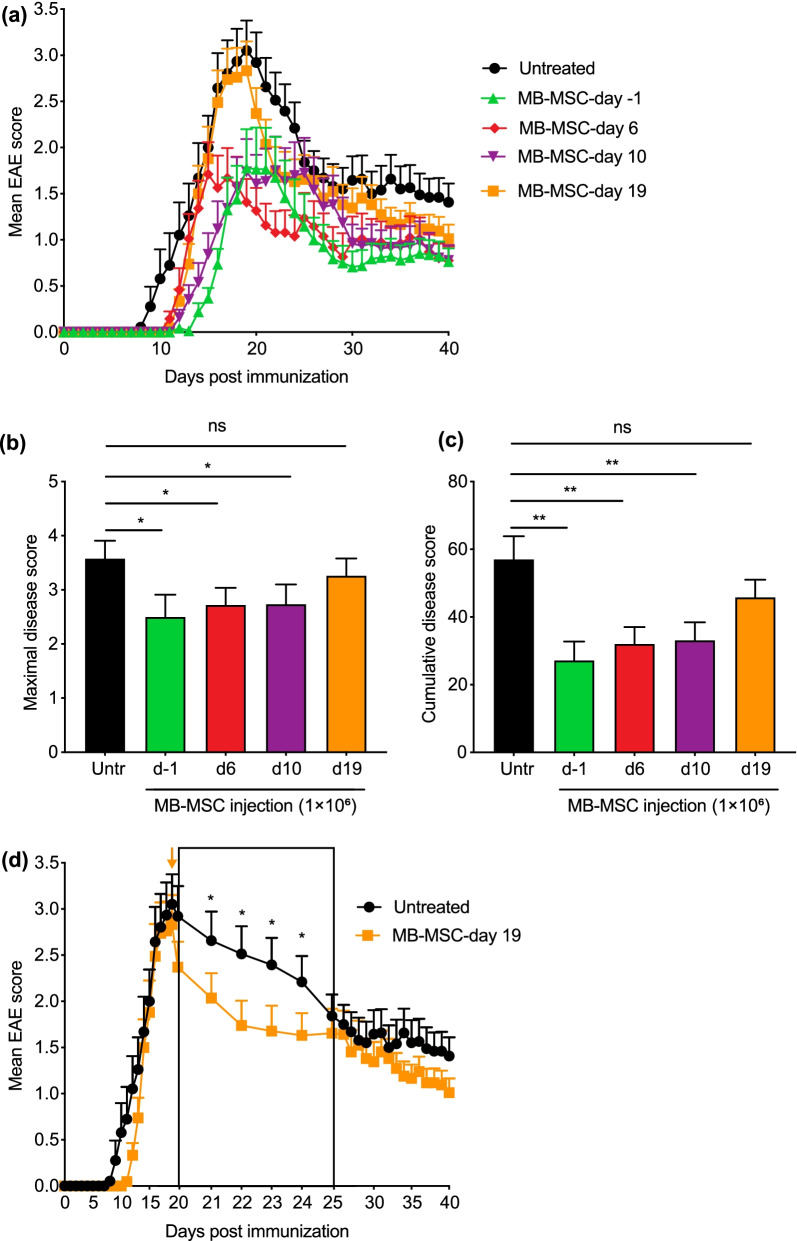
MB-MSCs ameliorate EAE when transplanted prior to and after disease induction. P4 MB-MSCs were transplanted via the tail vein (1 × 10^6^/mouse) either 1 day before EAE induction or 6, 10, or 19 days after EAE induction. Mice that received only PBS served as untreated controls. **a** EAE severity was monitored daily. **b**, **c** Maximal and cumulative disease scores were determined and compared to untreated controls. **d** In mice that received MB-MSCs on day 19, the disease severity was compared to untreated controls during the following 6 days. Pool of four experiments. *n* = 17, 19, or 21 per group

To evaluate whether MB-MSC transplantation could be used as a preventive therapy, MB-MSCs were transplanted one day before EAE induction (day − 1). To test therapeutic effectiveness, MB-MSCs were transplanted on day 10 when inflammatory T cells reached their maximal response, characterized by the highest accumulation of IFNγ- and IL-17-expressing CD4^+^ T cells in spleen and the start of immune cell infiltration into the CNS (Additional file 1: Fig. S1b, c), and on day 19 at the peak of the disease and of the CNS infiltration by immune cells (Additional file 1: Fig. S1c). MB-MSCs transplanted on day − 1 profoundly suppressed EAE development as maximal and cumulative disease scores were significantly reduced (Fig. 3a–c), indicating that MB-MSCs have the potential to prevent EAE. MB-MSCs transplanted on day 10 also displayed significant therapeutic benefits (Fig. 3a–c). Transplantation on day − 1 and day 10 achieved similar therapeutic effects as on day 6, although day − 1 transplantation tended to be slightly more beneficial. As expected, transplantation on day 19 neither influenced maximal disease scores (usually observed on day 19) nor cumulative disease scores. However, mice that received MB-MSCs on day 19 displayed a faster remission in the five days following transplantation (Fig. 3d). Thus, we demonstrated therapeutic benefits of MB-MSC transplantation at various stages of EAE.

### MB-MSCs have therapeutic effects comparable to UC-MSCs

**Fig. 4:**
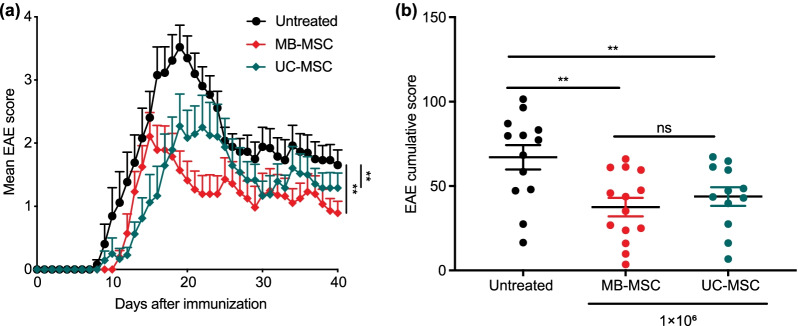
MB-MSCs have similar therapeutic effects on EAE as UC-MSCs. EAE was induced in mice and 6 days later, P4 MB-MSCs and P4 UC-MSCs were transplanted via the tail vein (1 × 10^6^/mouse). Mice that received only PBS served as untreated controls. **a** EAE disease severity was monitored daily and **b** cumulative disease scores were compared among the three groups. Pool of two experiments. *n* = 12, 13, or 14 per group

UC-MSCs have been clinically used as allo-MSCs and have been shown to restore neurological function [37]. We therefore compared the therapeutic efficacy of MB-MSCs to UC-MSCs by transplanting both types of MSCs six days after EAE induction. Both types of MSCs suppressed the development of EAE upon transplantation as shown by reduced cumulative disease scores (Fig. 4a, b). Notably, MB-MSCs displayed a therapeutic efficacy comparable to UC-MSCs without significant difference between these two groups (Fig. 4b). This suggests that MB-MSCs, a readily accessible source of MSC, have the potential to replace UC-MSCs as a therapeutic option in allotransplantation.

### MB-MSC transplantation into the peritoneal cavity is similarly effective as intravenous transplantation

**Fig. 5:**
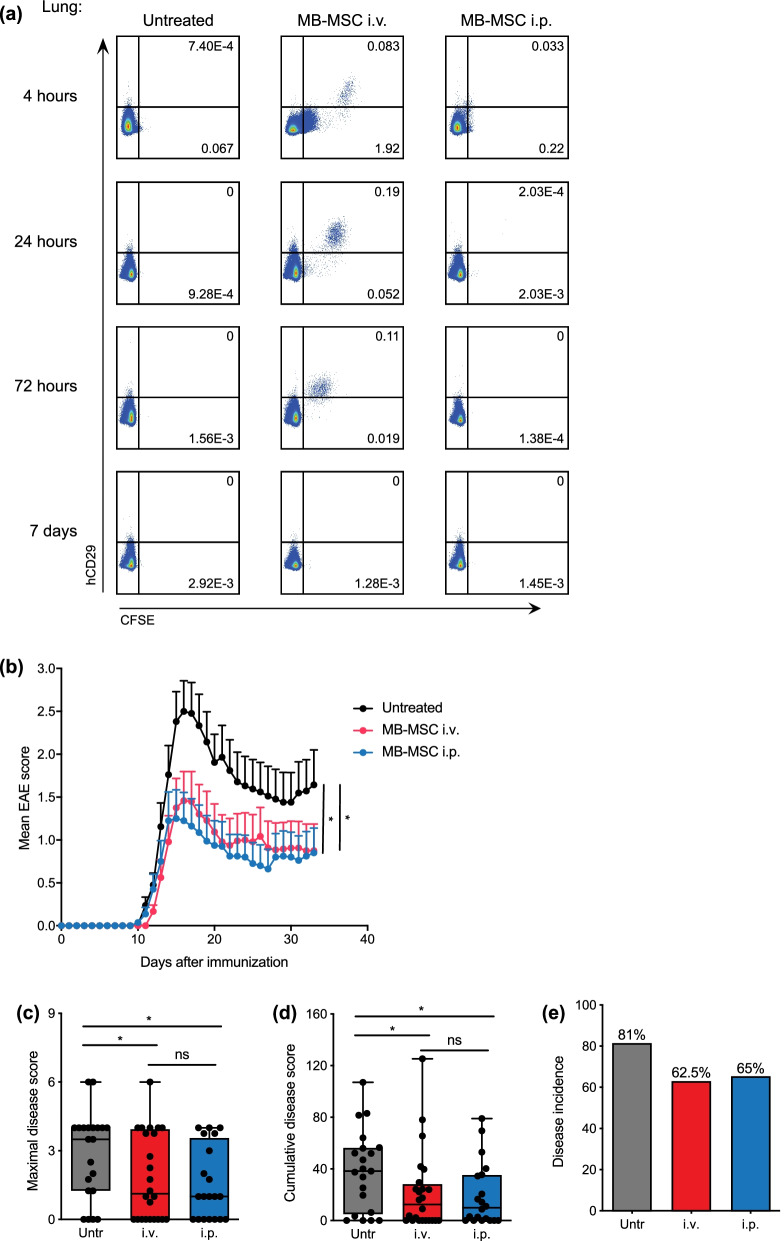
MB-MSC transplantation into the peritoneum reduced EAE disease severity as effective as intravenous transplantation. **a** P4 MB-MSCs were labelled with CFSE and transplanted by either i.v. (MB-MSC_i.v.) or i.p. (MB-MSC_i.p.) injection 6 days after EAE induction. 4, 24, 72 h and 7 days after transplantation, MB-MSCs were detected in the lungs by analyzing CFSE and human CD29 staining. Representative FACS plots demonstrate the presence of MB-MSCs in lung after i.v. injection but not i.p. injection. *n* = 2 per group and time point. **b** EAE severity was monitored daily in mice that received MB-MSC i.v. or i.p. or were left untreated. **c**–**e** Maximal disease scores, cumulative disease scores, and disease incidence rate were determined and compared. Pool of four experiments. *n* = 20, 21, or 24 per group

It is known that after intravenous injection, large amounts of stromal cells are retained in the lungs of the recipient mice. Some studies demonstrated that these cells embolized in the lungs are responsible for the improvement of myocardial infarction in mice [38]. To determine the importance of this mechanism in EAE, we conducted intraperitoneal (i.p.) injection to circumvent the lung retention of MB-MSCs. Whereas intravenously transplanted hCD29^+^ CFSE-labelled MB-MSCs accumulated in the mouse lungs, MB-MSCs were not detected in lungs when they were delivered into the peritoneal cavity (Fig. 5a). MB-MSCs injected i.p. had a similar protective effect as MB-MSCs injected i.v.: Both treatments achieved a comparable reduction of the maximal and cumulative disease scores and similarly reduced disease occurrence compared with the untreated group (Fig. 5b–e). Thus, lung retention of MB-MSCs is not essential to suppress the development of EAE.

### MB-MSCs transplanted i.v. or i.p. suppress Th1 and Th17 cell responses in peripheral lymphoid organs

**Fig. 6:**
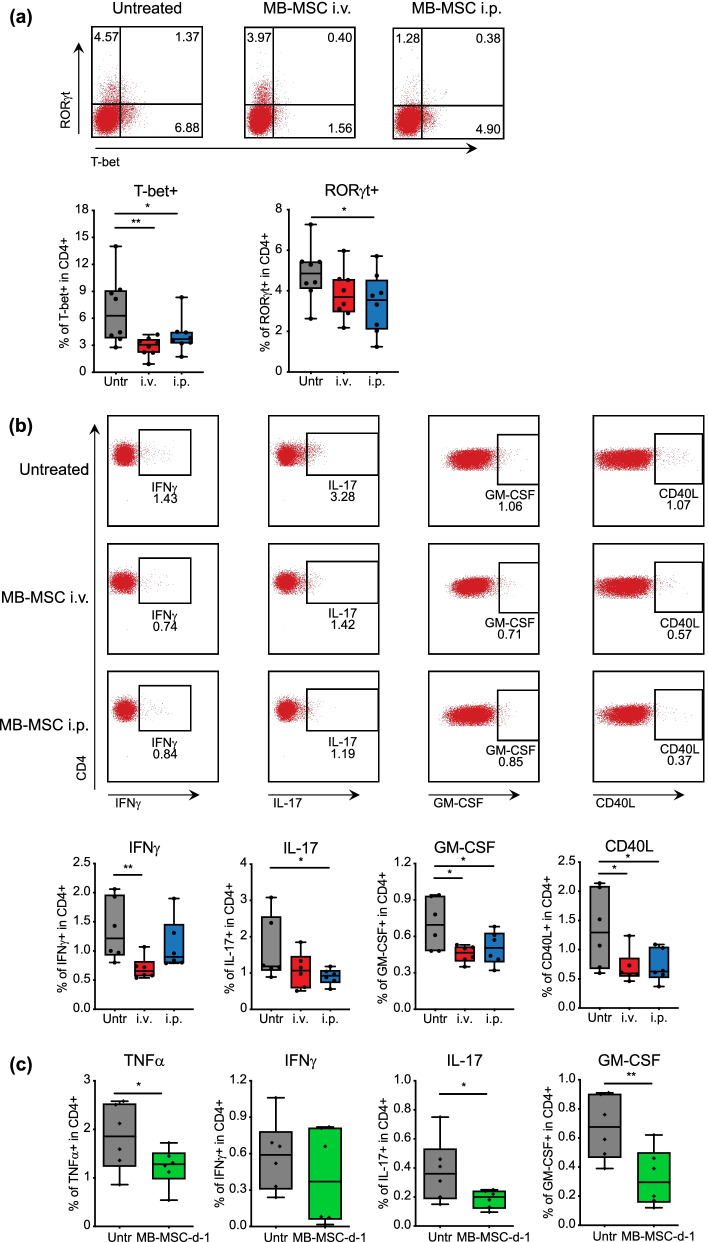
MB-MSCs transplanted i.v. or i.p. suppress Th1 and Th17 cell responses. EAE was induced in mice and 6 days later, P4 MB-MSCs were transplanted via i.v. (i.v.) or i.p. (i.p.) route. Mice that received only PBS served as untreated controls. On day 7, CD4 T cell responses were analyzed in spleen. **a** The transcription factors T-bet and RORγt were stained and percentages of T-bet and RORγt-expressing CD4^+^ cells were plotted and compared. Pool of four experiments with two mice per group per experiment (*n* = 8). **b** Intracellular cytokine staining was performed after MOG_35-55_ peptide restimulation, and percentages of IFNγ, IL-17, GM-CSF, and CD40L-expressing cells were plotted and compared. Pool of three experiments with two mice per group per experiment (*n* = 6). **c** Mice were transplanted i.v. with P4 MB-MSC or PBS. One day later, EAE was induced, and 5 weeks later, CD4^+^ T cell responses were analyzed in spleen after MOG_35-55_ peptide restimulation. Percentages of TNFα, IFNγ, IL-17, and GM-CSF-expressing cells were plotted and compared. Pool of three experiments with two mice per group per experiment (*n* = 6)

We next evaluated the in vivo T cell responses in peripheral lymphoid organs of MB-MSC-transplanted and untreated mice. Six days after EAE induction, we transplanted MB-MSCs via i.v. or i.p. injection and analyzed regulatory T cells (Treg) and inflammatory T cell responses in the spleen on the following day. In contrast to the Treg-inducing capacity of other types of MSCs [39, 40], MB-MSCs had no impact on the accumulation of Treg cells in spleen (Additional file 1: Fig. S2). However, Th1 and Th17 cells were reduced after MB-MSC transplantation via either route, as identified by their key transcription factors T-bet and RORγt, respectively (Fig. 6a). This observation was substantiated by intracellular cytokine staining assays in which the percentages of IFNγ-, IL-17-, and GM-CSF-expressing cells were reduced in the spleens of the MB-MSC-transplanted mice (Fig. 6b). Furthermore, transplanted MB-MSCs also significantly suppressed the expansion of MOG-reactive CD4 T cells identified by CD40L expression. To investigate whether the suppression of inflammatory T cell responses by transplanted MB-MSCs is a persistent effect, we transplanted MB-MSCs one day before EAE induction and evaluated the inflammatory T cell responses 5 weeks later. Importantly, CD4 T cells that expressed TNFα, IL-17, or GM-CSF remained significantly reduced in spleens of MB-MSC-transplanted mice (Fig. 6c). Thus, MB-MSCs can have a long-term therapeutic effect.

### Transplanted MB-MSCs suppress the activity of APCs

**Fig. 7:**
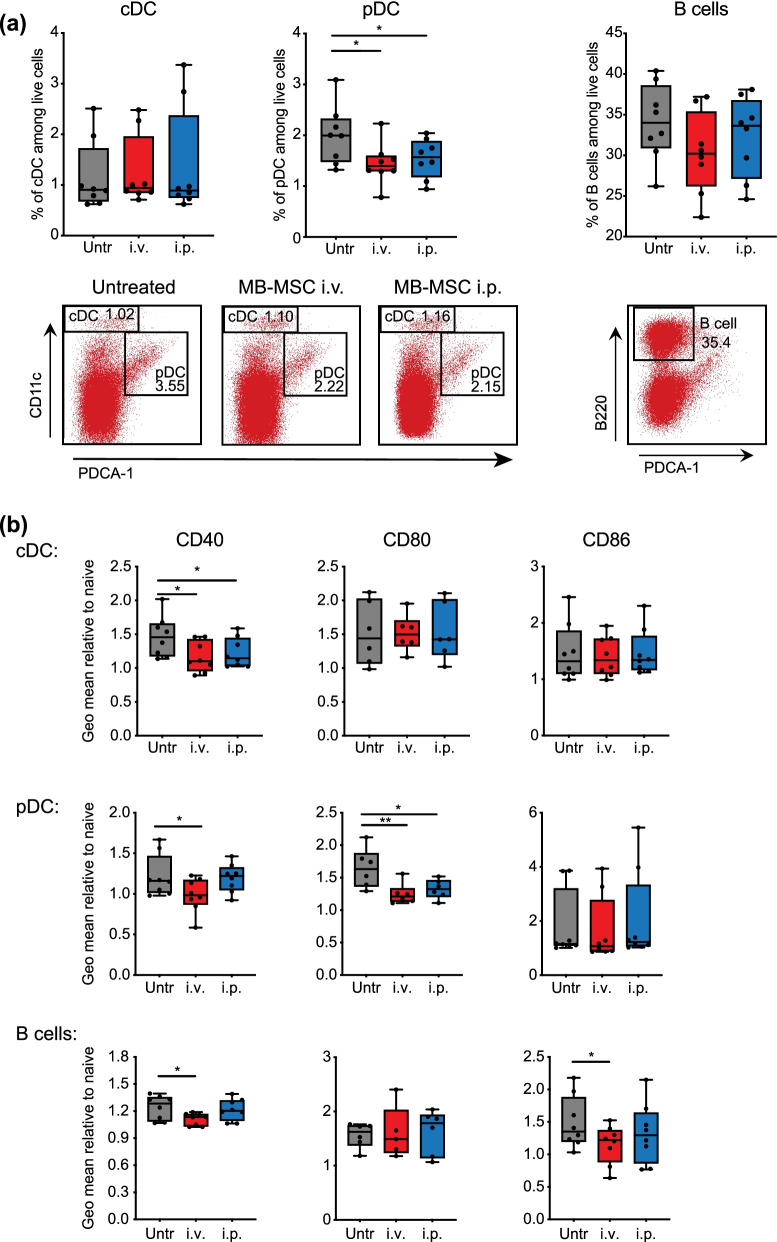
MB-MSCs transplanted i.v. or i.p. dampen the co-stimulatory potential of APCs. EAE was induced in mice and 6 days later, P4 MB-MSCs were transplanted via i.v. (i.v.) or i.p. (i.p.) route. Mice that received only PBS served as untreated controls. On day 7, various types of APCs were analyzed in spleen. **a** Percentages and representative FACS plots of cDC, pDC, and B cells were plotted. **b** The expression level of co-stimulatory molecules on these three types of APCs was plotted and compared. Pool of four experiments with two mice per group per experiment (*n* = 8)

To understand how transplanted MB-MSCs suppress the differentiation of inflammatory Th1 and Th17 cells in spleens, we investigated the antigen presentation and co-stimulatory capacity of APCs. First, we quantified APC populations in the spleen: The percentage of pDCs was significantly reduced in mice that had received MB-MSCs compared to untreated mice (Fig. 7a), while the percentages of B cells, monocytes, macrophages, and neutrophils were unchanged (Additional file 1: Fig. S3). Given the importance of pDCs in activating antigen-specific T cells during the development of EAE [41, 42], these data suggest that pDCs could be the intermediary for MB-MSCs to suppress EAE-related inflammation. We further determined the cell surface expression of the co-stimulatory factors CD80, CD86, CD40, and OX40L on APCs. We detected reduced expression of CD86 and CD40 on B cells, CD40 on cDCs, CD80 and CD40 on pDCs (Fig. 7b). Thus, our findings point to a general suppression of the activation status of APCs mediated by the transplantation of MB-MSCs. Collectively, these data suggest that transplanted MB-MSCs, through their suppression of APCs, restrict the activation of inflammatory T cells and thus protect mice from EAE.

## Discussion

Menstrual blood is a non-invasive reliable source of high-quality MSCs for allotransplantation with potential for cell-based therapy. Here we show that MB-MSCs ameliorated EAE severity upon transplantation at various time points prior to and after EAE induction. The EAE suppression by MB-MSCs was as effective as UC-MSCs. MB-MSC transplantation via i.p. or i.v. routes yielded similar EAE suppression. In both cases, it dampened the activation status of APCs and suppressed the inflammatory Th1 and Th17 cell responses (Graphical abstract).

In the last decade, 28 clinical trials using MSCs to treat MS have been conducted, of which 4 trials applied allo-MSCs from umbilical cord blood. No severe side effects were detected and some patients showed improved neurological parameters and increased walking time [43–45], demonstrating therapeutic effectiveness of allo-MSC transplantation. Although in one preclinical study transplanted BM-MSCs did not ameliorate EAE in mice [46] (possibly due to the usage of heparin-supplemented medium as control and as reconstitution solution for MSCs, since heparin is known to suppress EAE development [47, 48]), most of the studies have revealed beneficial effects of MSCs on EAE. Furthermore, xenotransplantation with human MSCs also displayed suppressive effects on EAE. When human BM-MSCs were transplanted into mice [49] or UC-MSCs transplanted into cynomolgus monkeys [50], the severity of clinical symptoms, immune cell infiltration, and demyelination were reduced. We show here similarly potent effects for MB-MSCs transplanted one day before and six, ten, or nineteen days after EAE induction. The protective effects of MB-MSCs transplanted prior to EAE induction provide an opportunity for a non-aggressive preventive therapy for high-risk individuals such as identical twins of MS patients, who have an about 30% increased chance of also developing MS [51, 52]. Therapeutic effects of MSCs are more desirable as transplantation could be used as a curative approach. Our data demonstrate that MB-MSCs can suppress EAE when transplanted on day six or ten after EAE induction. Even when transplanted on day 19, MB-MSCs provided a rapid improvement in the recovery phase in this model. Whether this translates into a potential therapeutic effect remains to be tested in detail and could be assessed in non-remitting chronic EAE disease models. Overall, we show that MB-MSCs achieved a similar protective effect as UC-MSCs and thus should be considered a candidate for ready-to-use allo-MSC products.

The administration route determines the microenvironment that MSCs first encounter in recipients and may thus influence their immunosuppressive mechanisms. Intravenous injection is most common due to its convenience; however, MSCs administered via this route are easily trapped in lung capillaries and thus may fail to enter the peripheral immune system [31]. Further, MSC injection via the i.v. route is accompanied by the risk of instant blood-mediated inflammatory reactions that compromise safety and therapeutic efficacy [3, 53, 54]. As an alternative systemic delivery, MB-MSCs can be transplanted via the i.p. route. In this way, the cells are not trapped in the lungs and do not cause hemocompatibility-related issues, while they still generated a comparable protective effect. The main mechanisms of action of MSCs in supporting tissue regeneration and immunomodulation are cell-contact-dependent or -independent mechanisms, mainly mediated via the secretion of trophic and immunomodulatory factors [3, 53, 55]. Hence, the fact that both i.v.- and i.p.-delivered MB-MSCs are therapeutically beneficial provides broader opportunities for MSC administration.

The exact mechanisms by which MB-MSCs mediate their beneficial outcomes have remained ill defined. Our data demonstrate an association of ameliorated disease with suppressed Th1 and Th17 cell responses in the periphery. Interestingly, MB-MSCs delivered i.v. tended to display a stronger suppression of Th1 responses, while MB-MSCs delivered i.p. featured a stronger suppression of Th17 responses. The MB-MSC-induced suppression of CD4 T cell responses was probably mediated by reduced APC activity: The presence of pDC in the spleen was significantly suppressed by both i.v.- and i.p.-delivered MB-MSCs. pDC plays an important role in initiating EAE via promoting the priming of Th1 and Th17 cells [41], so their numeric reduction could explain limited Th1 and Th17 responses in MB-MSC-transplanted mice. CD40 co-stimulation induces IL-12 production and results in the induction of Th1 responses [56, 57]. APCs, especially those of mice that had received MB-MSCs i.v., showed reduced CD40 expression. Thus, this lack of CD40 may provide a molecular explanation of the reduced Th1 response. In addition, surface expression of the co-stimulators CD80, CD86, and OX40L was also reduced on APCs of MB-MSC-transplanted mice.

## Conclusions

Human menstrual blood-derived MSCs, which can be isolated non-invasively and could be stored for acute application, ameliorated the disease severity of EAE upon transplantation, regardless of the route or the time of delivery, by suppressing T cell activation in peripheral lymphoid organs and immune cell infiltration into the CNS.

## Supplementary Information

**Additional file 1: Figure S1**. Kinetics of T cell responses in dLN and spleen, and immune cell infiltration in CNS during the course of EAE. EAE was induced in mice and immune cells were analyzed at various time points thereafter. (a, b) IFNγ- and IL-17-expressing CD4^+^ T cells in dLN and spleen were determined by FACS after MOG_35-55_ peptide restimulation. (c) CNS-infiltrating immune cells with high CD45 expression were determined by FACS ex vivo. Pool of two experiments with two mice per time point (*n* = 4 per time point). **Figure S2**. MB-MSC transplantation does not affect the frequency of regulatory T cells. EAE was induced in mice and 6 days later, P4 MB-MSCs were transplanted i.v.. Mice that received only PBS served as untreated controls. On day 7, the percentages of Foxp3-expressing cells among CD4 T cells in spleen were determined by FACS. Pool of four experiments with two mice per group per experiment (*n* = 8). **Figure S3**. MB-MSCs transplanted either i.v. or i.p. did not affect the accumulation and activation of macrophages, neutrophils, and monocytes. EAE was induced in mice and 6 days later, P4 MB-MSCs were transplanted via i.v. (i.v.) or i.p. (i.p.) route. Mice that received only PBS served as untreated controls. On day 7, the accumulation and activation of macrophages, monocytes, and neutrophils were analyzed in spleen. (a) Representative FACS plots and percentages of macrophages, monocytes, and neutrophils. (b) The expression level of costimulatory molecules on these three types of cells. Pool of four experiments with two mice per group per experiment (*n* = 8).

## Data Availability

The datasets in this study are available from the corresponding authors upon request.

## Abbreviations

MB-MSC: Menstrual blood-derived mesenchymal stromal cell
UC-MSC: Umbilical cord-derived mesenchymal stromal cell
allo-MSC: Allogeneic MSC
MS: Multiple sclerosis
EAE: Experimental autoimmune encephalomyelitis
CNS: Central nervous system
APC: Antigen-presenting cell
i.v.: Intravenous injection
i.p.: Intraperitoneally injection
