# Supplementary material for: Menstrual blood-derived mesenchymal stromal cells efficiently ameliorate experimental autoimmune encephalomyelitis by inhibiting T cell activation in mice
Source: Stem Cell Res Ther. 2022 Apr 11;13:155. doi: 10.1186/s13287-022-02838-8 (PMC8995916; doi:10.1186/s13287-022-02838-8)
Supplement: Supplementary file 1 — Additional file 1: Figure S1. Kinetics of T cell responses in dLN and spleen, and immune cell infiltration in CNS during the course of EAE. EAE was induced in mice and immune cells were analyzed at various time points thereafter. (a, b) IFNγ- and IL-17-expressing CD4+ T cells in dLN and spleen were determined by FACS after MOG35-55 peptide restimulation. (c) CNS-infiltrating immune cells with high CD45 expression were determined by FACS ex vivo. Pool of two experiments with two mice per time point (n = 4 per time point). Figure S2. MB-MSC transplantation does not affect the frequency of regulatory T cells. EAE was induced in mice and 6 days later, P4 MB-MSCs were transplanted i.v.. Mice that received only PBS served as untreated controls. On day 7, the percentages of Foxp3-expressing cells among CD4 T cells in spleen were determined by FACS. Pool of four experiments with two mice per group per experiment (n = 8). Figure S3. MB-MSCs transplanted either i.v. or i.p. did not affect the accumulation and activation of macrophages, neutrophils, and monocytes. EAE was induced in mice and 6 days later, P4 MB-MSCs were transplanted via i.v. (i.v.) or i.p. (i.p.) route. Mice that received only PBS served as untreated controls. On day 7, the accumulation and activation of macrophages, monocytes, and neutrophils were analyzed in spleen. (a) Representative FACS plots and percentages of macrophages, monocytes, and neutrophils. (b) The expression level of costimulatory molecules on these three types of cells. Pool of four experiments with two mice per group per experiment (n = 8). [file 13287_2022_2838_MOESM1_ESM.pdf]

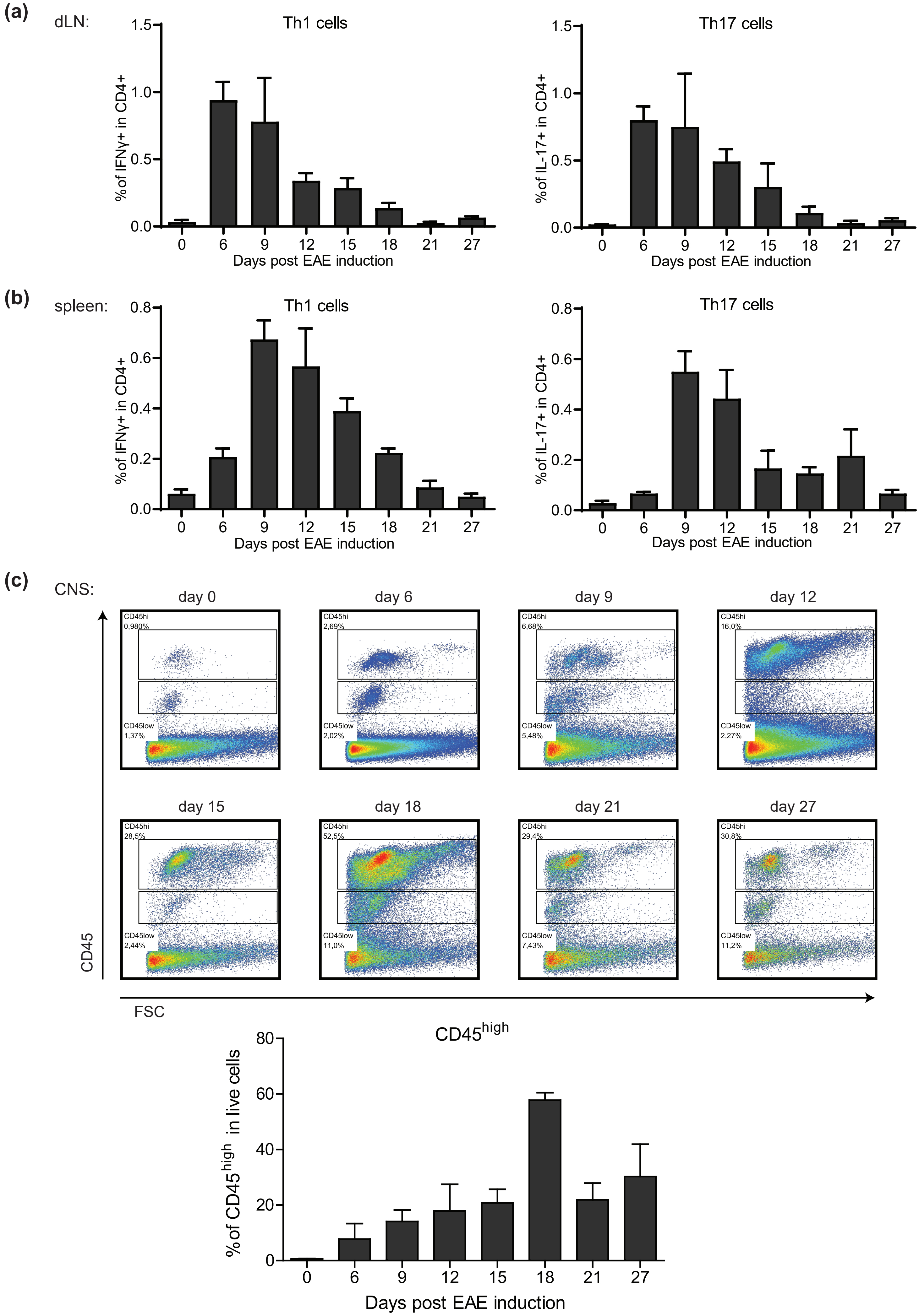

Supplementary figure 1. Kinetics of T cell responses in dLN and spleen, and immune cell infiltration in CNS during the course of EAE.

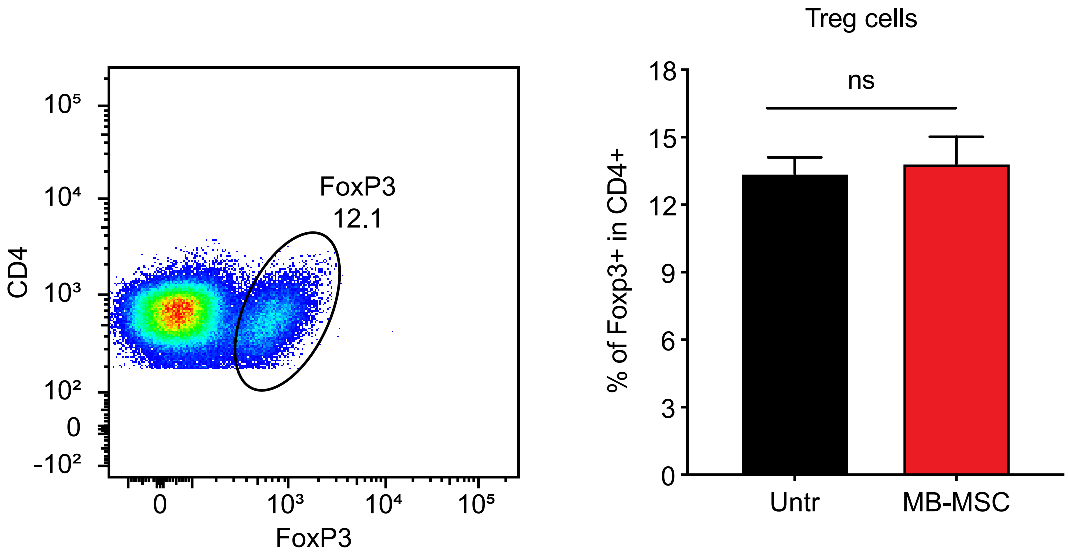

Supplementary figure 2. MB-MSC transplantation does not affect the frequency of regulatory T cells.

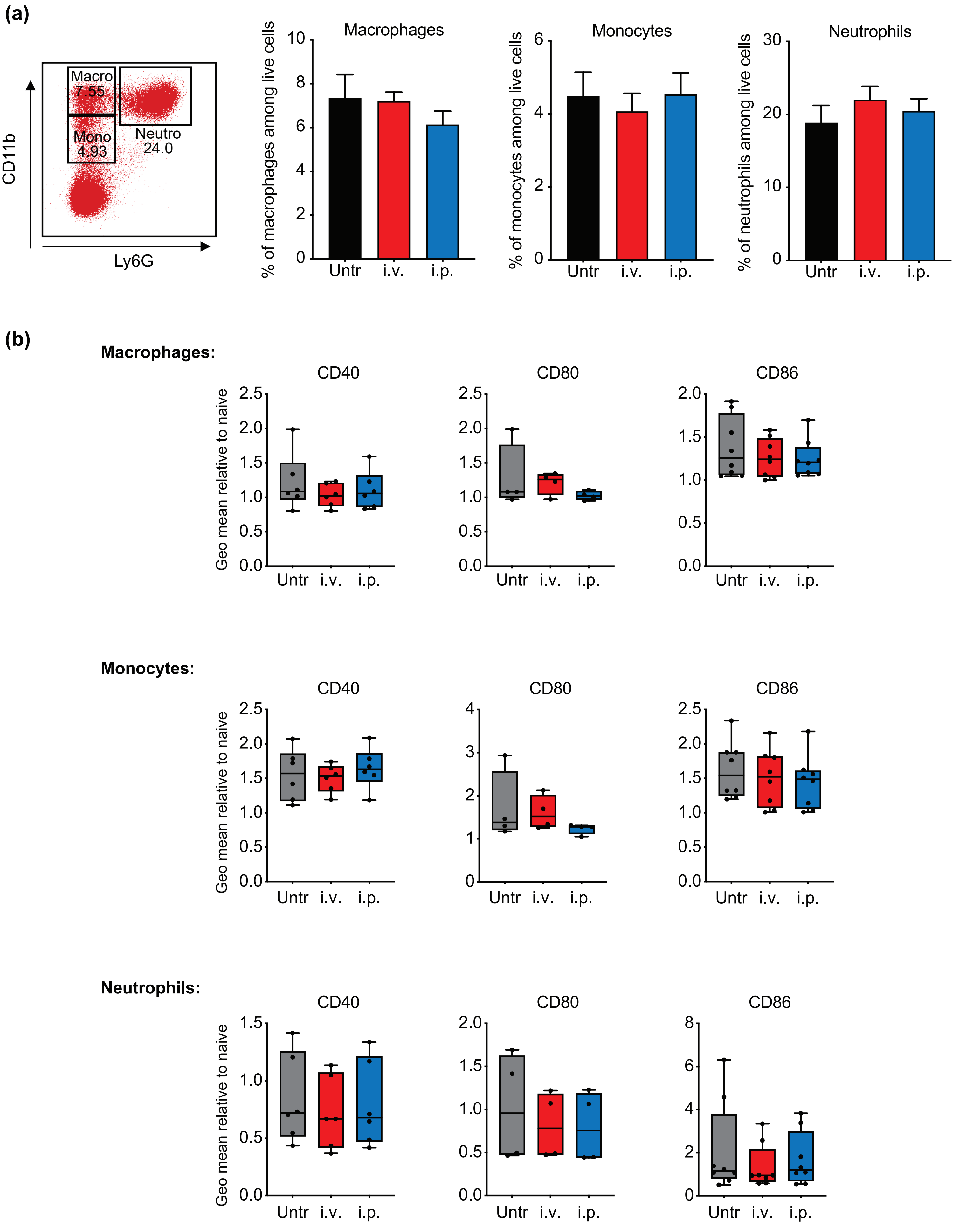

Supplementary figure 3. MB-MSCs transplanted either i.v. or i.p. did not affect the accumulation and activation of macrophages, neutrophils, and monocytes.
